# Wedge-Shaped Implants for Minimally Invasive Treatment of Narrow Ridges: A Multicenter Prospective Cohort Study

**DOI:** 10.3390/jcm9103301

**Published:** 2020-10-14

**Authors:** Tomaso Vercellotti, Giuseppe Troiano, Francesco Oreglia, Teresa Lombardi, Gianluca Gregorig, Emanuele Morella, Antonio Rapani, Claudio Stacchi

**Affiliations:** 1 Department of Surgical Sciences and Integrated Diagnostics, University of Genova, 16100 Genova, Italy; tomaso@vercellotti.com; 2 Department of Clinical and Experimental Medicine, University of Foggia, 71122 Foggia, Italy; giuseppe.troiano@unifg.it; 3 Private Practice, 37126 Verona, Italy; foreglia@yahoo.it; 4 Department of Health Sciences, Magna Græcia University, 88100 Catanzaro, Italy; drteresalombardi@libero.it; 5 Private Practice, 33012 Sappada (UD), Italy; gregoriggl@libero.it; 6 Private Practice, 20099 Sesto San Giovanni (MI), Italy; info@studiodrmorella.com; 7 Department of Medical, Surgical and Health Sciences, University of Trieste, 34129 Trieste, Italy; rapani.antonio@gmail.com

**Keywords:** wedge implant, narrow ridge, piezosurgery, atrophic ridge, minimally invasive, patient-related outcomes

## Abstract

The present study aims to investigate clinical and patient-centered outcomes after the implant-supported rehabilitation of narrow ridges using a novel wedge-shaped implant. Forty-four patients were treated with the insertion of 59 tissue-level wedge implants (1.8 mm bucco-lingual width) in horizontally atrophic ridges (mean bone width 3.8 ± 0.4 mm). The main outcome measures were: implant stability quotient (ISQ), marginal bone loss (MBL) and patient morbidity. Fifty-eight implants were functioning satisfactorily after one year of loading (98.3% survival rate). ISQ values measured in the mesio-distal direction resulted significantly higher than those in the bucco-lingual direction at all time points (*p* < 0.001). Both mesio-distal and bucco-lingual ISQ values at 6-month follow-up resulted significantly higher than at 4-month follow-up (*p* < 0.001 for both). Mean MBL was 0.38 ± 0.48 mm at prosthesis delivery (6 months after implant insertion) and 0.60 ± 0.52 mm after one year of functional loading. The majority of patients reported slight discomfort related to the surgical procedure. Postoperative pain score was classified as mild pain on the day of surgery and the first postoperative day and no pain over the following five days. Within the limitations of the present study, the device investigated showed low morbidity and positive short-term clinical results in narrow ridges treatment.

## 1. Introduction

The progressive resorption of the alveolar ridge after tooth extraction may lead to different degrees of jaw atrophy, resulting in reduced horizontal or vertical dimensions of the bone crest or in a combination of the above [1]. Insufficient bone volume can hinder implant therapy by causing two main problems: impossibility to insert the fixture in an adequate bone envelope and difficulty to place the implant in an optimal prosthetic position. The use of implants of reduced diameter has been proposed to overcome this situation. However, recent meta-analyses showed that only implants with a diameter ≥3 mm demonstrated no difference in survival rate compared to standard diameter implants (3.75–4 mm) [2,3]. Therefore, when advanced horizontal resorption of the alveolar ridge is present (width < 5 mm), bone regeneration procedures are frequently required for successful implant-supported rehabilitation [4]. Various surgical techniques (guided bone regeneration, bone blocks, ridge expansion) [5,6,7,8] have been proposed to increase crestal bone width and implants inserted into augmented sites have shown functional and aesthetic satisfactory long-term results [9]. However, regenerative approaches require advanced surgical skills and are associated with increased morbidity, prolonged duration of therapy and high costs [10].

Blade implants were proposed by Leonard Linkow in 1968 to overcome the limitations of root-form implants in atrophic ridges with reduced horizontal width [11]. Blade implants presented various designs generally characterized by a rectangular shape with two flat surfaces facing buccal and lingual corticals and one or more abutments originating from the thin coronal section of the plate. These implants showed a high failure rate [12,13] due to unpredictability in achieving primary stability, the type of loading protocol and postoperative complications. Some papers reported encouraging clinical results of blade implants after more than 20 years of service [14,15], but their use in daily practice progressively declined over time. In 2014, however, based on new information, the Food and Drug Administration reclassified blade-form endosseous dental implants from class III (devices presenting potential high risk of illness or injury and needing pre-market submission for approval) to class II (low risk devices without the need of pre-market submission) [16]. This measure paved the way for the introduction of the clinical use of novel implant shapes deriving from the blade implant concept, but exploiting current knowledge significantly improved the implant site preparation technique, surface treatment and prosthetic protocols.

The aim of the present multicenter prospective cohort study is to evaluate clinical effectiveness and patient-centered outcomes after implant-supported rehabilitation of edentulous ridges with reduced horizontal width using a recently designed wedge-shaped implant.

## 2. Materials and Methods

### 2.1. Patient Selection

Any partially edentulous patient requiring implant-supported rehabilitation, being 18 years or older, and able to understand and sign a written informed consent form was eligible for this multicenter prospective cohort study. The present study was designed as a pragmatic trial in order to better reflect clinical reality. Broad inclusion criteria were applied, including any type of bone and location, different prosthetic rehabilitations and smokers.

The following inclusion criteria were adopted:

- Bone height in the programmed implant site ≥10 mm and bone width comprised between 3.5 and 5 mm (measured 1 mm below the most cranial point of the alveolar crest);
- Healed bone crest (almost three months elapsed after extraction or tooth loss);
- Patient age >18 years;
- Patients able to examine and understand the study protocol.

The following exclusion criteria were adopted:

- General contraindications to implant surgery;
- Immunosuppressed or immunocompromised;
- Irradiated in head and neck area within the past five years;
- Treated or under treatment with intravenous antiresorptives;
- Uncontrolled diabetes (glycated hemoglobin ≥7.5%);
- Pregnant or lactating;
- Poor oral hygiene or motivation (full mouth plaque score >30% and full mouth bleeding score >20%);
- Untreated periodontitis;
- Alcohol or drugs abuse;
- Psychiatric problems or unrealistic expectations;
- Participating in other studies, if the present protocol could not be properly followed.

The principles outlined in the Helsinki Declaration on clinical research involving human subjects, as revised in Fortaleza (2013), were adhered to. All patients were thoroughly informed regarding the scope and protocol of the study (including clinical procedures, follow-up evaluations, and any potential risks involved). Patients were allowed to ask questions pertaining to this study, were informed of treatment alternatives and finally signed an informed consent form. The study protocol was approved by the relevant Ethical Committee (Regione Calabria, Sezione Area Nord, No. 1/2017) and recorded in a public register of clinical trials (www.clinicaltrials.gov—NCT03290729).

Preoperative radiographs (periapical and Cone Beam Computed Tomography—CBCT), together with clinical examination were used to assess available bone volume of edentulous sites and a prosthodontic evaluation was performed to define treatment planning. Patients were treated in six private clinical centers by experienced operators (T.V.; F.O.; T.L.; G.G.; E.M. and C.S.) and all follow-up visits were performed at the respective treatment centers.

**Figure 1:**
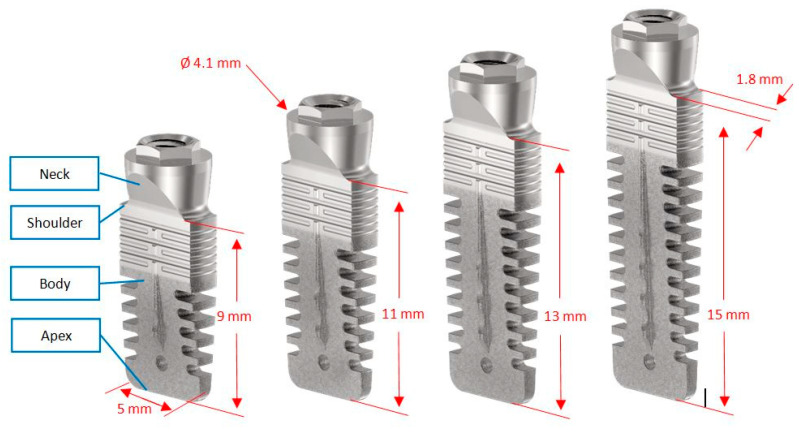
Device investigated: a tissue level, wedge-shaped dental implant with an external connection and four different lengths.

The device investigated was a commercially available, tissue-level, titanium grade 23 [17], press-fit dental implant with an external connection (Rex TL, Rex Implants Inc., Columbus, OH, USA). This implant, measuring 5 mm in the mesio-distal direction and 1.8 mm in the bucco-lingual direction, is characterized by a wedge shape (Figure 1) and a hybrid surface treatment (minimally rough in the coronal part, moderately rough in the middle and apical parts) [18]. Operators were free to choose implant length (9, 11, 13 or 15 mm) according to clinical indications and their own expert opinions.

### 2.2. Surgical and Prosthetic Procedures

**Figure 2:**
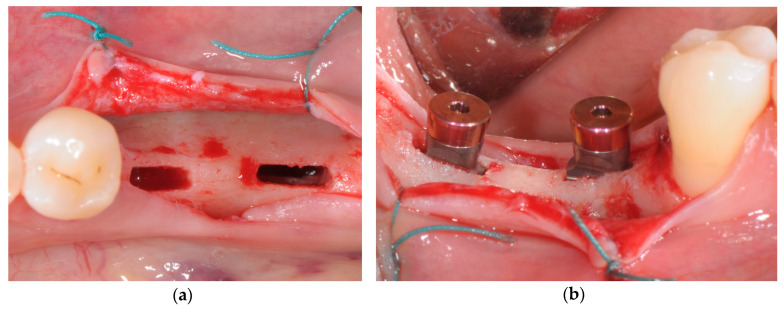
(**a**) Implant site preparation performed with piezoelectric tips; (**b**) wedge implants seating in the final position.

Antibiotic prophylaxis was prescribed (amoxicillin 2 g one hour prior to surgery or clindamycin 600 mg if allergic to penicillin). Patients were asked to rinse with chlorhexidine digluconate solution (0.2%) for 1 min approximately 10 min before surgery. Under local anesthesia, a minimally invasive full-thickness flap was elevated. Implant site preparation was entirely performed using a piezoelectric device (Piezosurgery Touch, Mectron, Carasco, Italy), following a standardized sequence of ultrasonic tips as recommended by the implant system manufacturer (Rex Implants, Colombus, OH, USA). Wedge-shaped implants (Rex TL, Rex Implants, Columbus, OH, USA) were press-fitted into the osteotomies using a magnetic mallet (IPD, Rex Implants, Columbus, OH, USA), until final seating was reached (Figure 2).

Surgical time (in minutes) from the first soft tissue incision to the moment at which the implant reached the final position was recorded for each implant.

Immediately after implant placement, an aluminum transducer (Smartpeg Type 4, Osstell, Göteborg, Sweden) was screwed into the implant and torqued to 15 Ncm to measure resonance frequency analysis (RFA). A blinded clinical assistant for each center recorded in duplicate implant stability quotient (ISQ) values from mesio-distal, disto-mesial, bucco-lingual and linguo-buccal directions using an integrated Osstell module (Implantmed, W&H, Burmoos, Austria). Instrument calibration was verified before and after each patient visit, using an implant fixed in an epoxy resin block. A cover screw was then connected to the implant and soft tissues were sutured around it for non-submerged healing using non-resorbable synthetic monofilament. Digital periapical radiographs were then performed using a long-cone paralleling technique with a Rinn-type film holder. The following exposure parameters were set: 65–90 kV, 7.5–10 mA and 0.22–0.25 s.

Patients were prescribed with analgesics when needed (paracetamol 500 mg tablets) and oral rinses of 0.12% chlorhexidine digluconate for 15 days following surgery.

Sutures were removed after 14 days and patients were recalled once every month to check the course of healing. RFA measurements were performed for each implant using the previously described protocol after 4 and 6 months of healing.

Impressions were taken 6 months after implant insertion and fixed screw retained metal-ceramic restorations (single crowns or short-span bridges) were delivered. A periapical radiograph was performed following the previously described protocol at prosthesis delivery to ensure the correct fitting of the framework. Patients then followed a maintenance program and were recalled every four months to control oral hygiene and periodontal conditions.

One year after prosthetic loading, implants were clinically evaluated and an additional periapical radiograph was performed.

### 2.3. Radiographic Measurements

**Figure 3:**
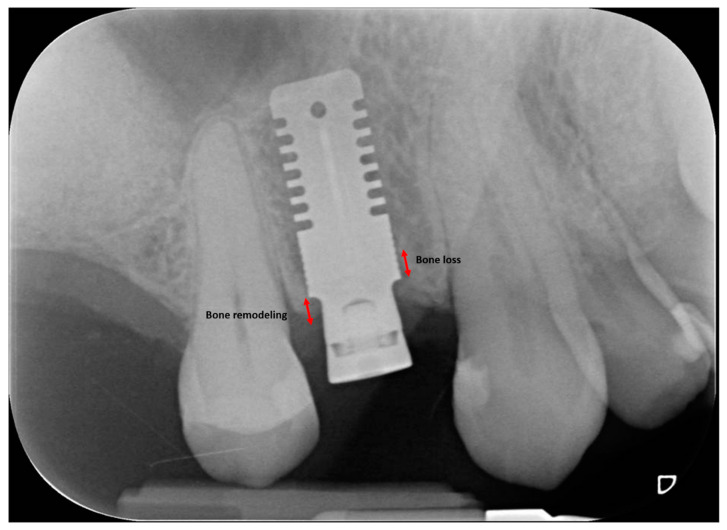
Peri-implant bone resorption is defined as bone loss when occurring apically to the implant shoulder, whereas it is defined bone remodeling when occurring coronally to the implant shoulder.

Marginal bone loss (MBL) was measured on both mesial and distal aspects of the implant as the linear distance between two points, the most coronal point of the implant shoulder and the most coronal bone-to-implant contact, corrected referring to the known height and diameter of each implant. A positive value was assigned when the bone crest was coronal to the implant shoulder, whereas a negative value was assigned when the bone crest was apical to the implant shoulder. According to Linkevicius et al. [19], peri-implant bone resorption is defined as bone loss when occurring apically to the implant shoulder, whereas it is defined as bone remodeling when occurring coronally to the implant shoulder (possible only for implants with the shoulder placed subcrestally at baseline) (Figure 3).

Radiographs showing evident deformation, poor image quality or other problems were immediately repeated. All radiographic measurements were made by a blinded calibrated examiner (A.R.), using a 30-inch led-backlit color diagnostic display and measuring software (Image J, National Institutes of Health, Bethesda, MD, USA). Each measurement was repeated three times at three different time points as proposed by Gomez-Roman and Launer [20]. Examiner calibration was performed by assessing ten radiographs, with a different author (C.S.) serving as a reference examiner. Intra-examiner and inter-examiner concordances were 90.7% and 84.8%, respectively, for linear measurements within ±0.1 mm.

### 2.4. Patient-Centered Outcomes

A specific form was delivered to the patient after surgery to collect patient-related outcomes.

The following information was recorded:

- Intra-operative discomfort perceived by the patient (VRS_discomfort_): recorded immediately after surgery on a 5-point visual rating scale (VRS) ranging from “0—no discomfort” to “4—very severe discomfort”;
- Pain perceived by the patient (VAS_pain_): recorded daily (evening) for 7 days following surgery on a 100-mm visual analogue scale (VAS) (ranging from “no pain” to “intolerable pain”). The patient was asked to place a line perpendicular to the VAS line at the point representing pain intensity;
- Use of painkillers (i.e., the number of paracetamol 500 mg tablets) assumed by the patient up to the 6th postoperative day.

### 2.5. Statistical Analysis

Statistical analysis was performed using the software Stata 16.1 (StataCorp, College Station, TX, USA), setting the threshold of statistical significance at α = 0.05. The implant was considered as the statistical unit. Prior to the present study, no clinical data on this specific implant were available in literature to perform a reliable sample size calculation. Differences in ISQ and MBL values between the different time points were analyzed by means of a Wilcoxon test, while the Mann–Whitney test was used to assess comparisons in ISQ and MBL between the different sites considered (mesio/distal and bucco/lingual). Differences in the duration of surgery and in VAS_pain_ among the six centers were evaluated by means of Kruskal–Wallis test followed by Bonferroni post-hoc test for multiple comparisons. In addition, univariate analysis for the association of different clinical variables with MBL was analyzed building multilevel linear models with random slopes.

## 3. Results

### 3.1. Study Population

From a total of 86 patients evaluated for entry to this study, 44 consecutive patients (15 male, 29 female, mean age 59.5 ± 12.0 years) fulfilled all inclusion criteria and were enrolled and treated in six centers from May 2017 to October 2018 with the insertion of 59 implants (14 implants in maxilla and 45 in mandible; 19 implants with 9 mm length and 40 implants with 11 mm length). One patient received three implants and 12 patients received two implants, while the remaining patients received one single implant. Focusing on the site of implant placement, 29 implants (49.15%) were placed at a premolar site, 25 (42.37%) at a molar site and 5 (8.47%) in the anterior area. No dropouts occurred during the entire follow-up period of the study. The main baseline patient and intervention characteristics are presented in Table 1.

### 3.2. Duration of the Surgical Procedure

The mean duration of the surgical procedure was 25.8 ± 13.6 min (range 11–105). No significant differences in surgical time were demonstrated among the six centers after Kruskal–Wallis test (*p* = 0.11).

### 3.3. Clinical Outcomes

The mean bone width measured 1 mm below the most cranial point of the alveolar crest was 3.8 ± 0.4 mm. No surgical complications were observed. One mandibular implant (9 mm length) was lost one month after insertion, whereas 58 implants were functioning satisfactorily one year after prosthetic loading (98.3% survival rate). No biological or mechanical complications were recorded.

**Figure 4:**
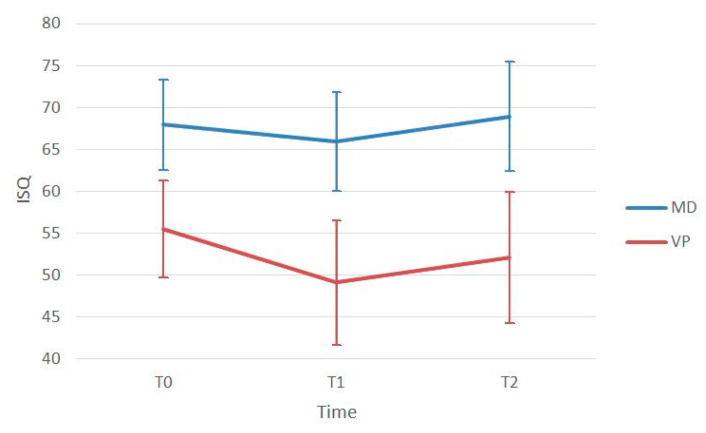
Implant stability pattern at baseline (T0), after 4 (T1) and 6 (T2) months of healing. ISQ: implant stability quotient; MD: mesio-distal direction; VP: vestibular-palatal direction.

The mean ISQ at implant insertion (ISQ T0) was 68.0 ± 5.4 in the mesio-distal direction and 55.5 ± 5.8 in the bucco-lingual direction. After four months of healing (ISQ T1), the mean ISQ was 65.9 ± 5.7 in the mesio-distal direction and 49.1 ± 7.5 in the bucco-lingual direction. After six months of healing (ISQ T2), the mean ISQ was 68.9 ± 6.5 in the mesio-distal direction and 52.1 ± 7.8 in the bucco-lingual direction (Table 2). The Mann–Whitney test showed that ISQ values measured in the mesio-distal direction resulted significantly higher than those in bucco-lingual direction at all time points (*p* < 0.001). A Wilcoxon test showed that both mesio-distal and bucco-lingual ISQ values at 6-month follow-up (ISQ T2) resulted significantly higher than at 4-month follow-up (ISQ T1) (*p* < 0.001 for both). No significant differences were found between ISQ values in maxilla and in mandible at T0 and T1, whereas ISQ resulted significantly higher in maxilla than in mandible at T2 (*p* = 0.04). A complete listing of ISQ measurements is reported in Table 2, and a stability pattern over time is depicted in Figure 4.

The mean MBL from T0 was 0.38 ± 0.48 mm at prosthesis delivery (6 months after implant insertion—T1) and 0.60 ± 0.52 mm after one year of functional loading (T2). A Wilcoxon test demonstrated significant differences in marginal bone levels between T0 and T1 and between T0 and T2 (*p* < 0.001), similar results were obtained for the analysis of marginal bone level changes between T1 and T2 (0.20 ± 0.19 mm, *p* < 0.001). Complete MBL measurements are listed in Table 3.

The univariate analysis of baseline variables possibly influencing MBL is shown in Table 4. No statistically significant relationships were demonstrated between MBL and patient age, gender, smoking habits, history of periodontal disease or implant length. Only mandibular implant locations were associated to increased peri-implant bone loss after one year of functional loading (*p* = 0.044).

### 3.4. Patient−Centered Outcomes

VRS_discomfort_ was 0 (no discomfort) in 12 patients, 1 (slight discomfort) in 18 patients, 2 (mild discomfort) in 12 patients and 3 (severe discomfort) in 2 patients. No patient reported very severe discomfort (VRS_discomfort_ = 4). The median VRS_discomfort_ was 1.

**Figure 5:**
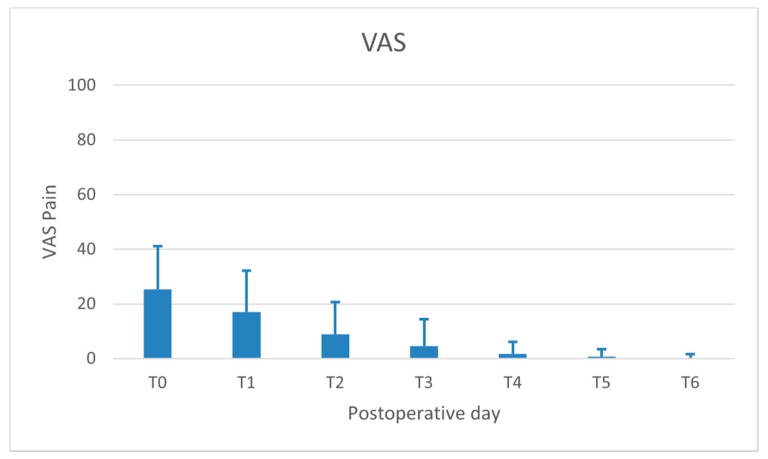
Trend of subjective pain assessed by visual analogue scale (VAS) on the day of surgery (T0) and the following six postoperative days.

The highest level of VAS_pain_ was recorded on the day of surgery (mean 24.0 ± 15.0 on a scale ranging from 0 to 100) and significantly decreased over time, up to 6 days after surgery. The trend of VAS_pain_ is depicted in Figure 5.

A small number of paracetamol tablets were assumed by all patients after the intervention (median = 1 tablet for the day of surgery and day 1; median = 0 for the following five days).

## 4. Discussion

An adequate bone envelope is a crucial pre-requisite for the long-term success of dental implants. In particular, a significant association has been demonstrated between buccal bone thickness at implant placement (<1.8–2 mm) and the incidence of bone dehiscence and mucosal recession over time [21,22,23]. However, radiographic studies demonstrated that roughly 90% of edentulous ridges, either in maxilla or in mandible, present insufficient width at crestal level to allow for ideal positioning of a standard diameter implant (3.75–4.0 mm) [24,25,26]. In the majority of cases, the therapeutic project should include horizontal augmentation procedures, use of narrow implants or a combination of the above. The most common horizontal augmentation techniques (ridge expansion, bone blocks and guided bone regeneration) are safe and effective but inevitably imply higher morbidity, prolonged therapy time and increased economic costs.

The present prospective cohort study demonstrates the feasibility of using wedge-shaped implants (1.8 mm width) for the rehabilitation of narrow ridges (mean 3.8 mm), without the association of any regenerative procedure. The survival rate at one-year follow-up was 98.3%. Similar outcomes were reported by systematic reviews investigating standard implants inserted after horizontal bone augmentation procedures conducted using different combinations of bone replacement grafts and barrier membranes. Donos et al., 2008, reported implant survival rates of 87% to 95% for the simultaneous approach and 99% to 100% for the staged approach [27]. Kuchler and von Arx, 2014, and Sanz-Sánchez et al., 2015, also reported high survival and success rates (>95%) for implants placed in regenerated sites [9,28].

Implant primary stability plays a fundamental role especially in the osseointegration of tissue level implants. During unsubmerged healing, the implant neck may be easily solicited by occlusal forces and/or by tongue and lip movements resulting in excessive micromotion potentially jeopardizing the osseointegration process [29,30]. Early animal experiments conducted using blade implants by Brunski et al., 1979, and Lum et al., 1991, showed that micromotions during the healing phase favored fibrous tissue interposition at the bone–implant interface [31,32]. This occurrence was relatively frequent in blade implants, where the implant bed was traditionally prepared by using fissure burs mounted on a high-speed handpiece and advanced surgical skills were necessary to obtain adequate primary stability predictably. In the present study, a good primary stability was obtained in all cases (mean ISQ at baseline 68.0 ± 5.4 in mesio-distal direction and 55.5 ± 5.8 in the bucco-lingual direction). Implant osteotomy for wedge implants was performed using a piezoelectric device, allowing for enhanced surgical control thanks to the microvibrations of the working tips and resulting in a predictable precise adaptation of the implant into the prepared site [33,34]. Additionally, a recent meta-analysis with trial sequential analysis showed that ultrasonic implant site preparation has a positive influence on early bone healing, resulting in faster development of secondary stability when compared to conventional drilling techniques [35]. It is interesting to note how the unconventional shape of the tested implant affected implant stability measurements. ISQ values were always significantly higher in the mesio-distal and disto-mesial directions than in the bucco-lingual or linguo-buccal directions.

In the present investigation, the authors observed that implant stability significantly decreased from T0 (baseline) to T1 (four months after surgery), returning to baseline levels only six months after surgery (T2). This slow increase in secondary stability could be explained by the fact that implants were inserted in atrophic ridges (mean bone width 3.8 mm), with most of the implant surface facing only buccal and lingual cortical bone. Recent histomorphometric and biomolecular studies clearly demonstrated that new bone formation starts primarily in the spongious bone and that the rate of new bone formation is significantly increased in trabeculated cancellous bone osteotomies compared to equivalent osteotomies in densely compact cortical bone [36]. These considerations could explain why, in the present study, ISQ values at T2 resulted significantly higher in maxilla than in mandible and suggest that the healing period after wedge implant placement in narrow ridges could be slightly longer than that of a standard implant inserted in a bone crest with a good quantity of spongious bone.

The mean early marginal bone loss (MBL) after one year of loading was 0.6 ± 0.52 mm as a result of various concurring factors (surgical trauma, biological width establishment, adaptive modifications to occlusal loading) [37,38]. The greatest amount of bone loss (mean 0.38 ± 0.48 mm) occurred during the first six months of healing, in perfect agreement with previous studies conducted on standard implants [38,39,40,41]. It is noteworthy that mean MBL around wedge implants is similar or lower than mean MBL reported in other studies for standard implants inserted in combination with horizontal augmentation procedures (guided bone regeneration, bone block augmentation, split crest) [7,42,43,44]. Among the baseline variables, univariate analysis suggested that implants inserted in the mandible are prone to lose more marginal bone than maxillary implants after one year of functional loading. This finding is in accordance with previous studies on narrow ridges treated with ridge expansion, showing that marginal bone loss is higher as bone density increases [45]. An increase in Young’s modulus and a decrease in cortical bone thickness resulted in elevated stresses under functional loading within both cancellous and cortical bone, possibly leading to marginal bone resorption [46].

Slight intraoperative discomfort was experienced by the majority of patients during wedge implant insertion, likely due to the short mean duration of surgery (25 min), similar to standard implant positioning. Standard implant surgeries are usually related to low pain scores: Kim et al. [47] demonstrated that postoperative pain and discomfort are proportional to the duration of the surgical procedure and the number of implants placed. The mean VAS_pain_ score recorded in the present study is similar to those recorded in other studies investigating single standard implant insertion with drilling [48,49] or ultrasonic technique [50]. In accordance with these outcomes, VAS_pain_ score was classified as mild pain on the day of surgery and the first postoperative day and no pain over the following five days. This tendency was confirmed by the extremely low quantity of rescue analgesic medication taken by the patients during the postoperative period. On the contrary, when advanced regenerative procedures are performed to augment crestal bone width, morbidity and patient discomfort always increase significantly. This is likely due to the prolonged surgical time, extensive detachment of muco-periosteal flaps and periosteal/muscular releasing incisions to attain primary closure of the surgical wound. In a study by Pistilli et al., 2014, 50% of patients still reported moderate pain 10 days after autogenous bone block augmentation [51]. A recent multicenter study on implants inserted with simultaneous guided bone regeneration showed VAS_pain_ scores higher than those reported in the present study during the entire week after surgery [52].

Main limitations of this prospective cohort study are the short follow-up and limited sampling. Important variables such as biological and mechanical failures, patient satisfaction and aesthetic concerns will be investigated in the 3- and 5-year post-loading follow-up of the present study. Moreover, adequately powered randomized clinical trials comparing wedge implants with horizontal augmentation procedures in the treatment of narrow ridges should be implemented. Regarding the generalization of the study outcomes, the device investigated has been tested in authentic clinical conditions with broad inclusion criteria and six different operators. Results, therefore, could be generalized with confidence to populations with similar characteristics.

## Figures and Tables

**Table 1:** Baseline patient and intervention characteristics.

Patients/Implants	44/59
Age (mean ± SD)	59.5 ± 12.0
Gender	15 male/29 female
Smoking	31 no smoker/13 smoker
History of Periodontitis	25 no/19 yes
Implant Location	14 maxilla/45 mandible
Implant Length	9 mm (19)/11 mm (40)

Age is expressed in years; SD: standard deviation.

**Table 2:** Mean implant stability quotient (ISQ) measurements at the different time points.

	ISQ T0	ISQ T1	T1-T0 (*p*-Value)	ISQ T2	T2-T0 (*p*-Value)	T2-T1 (*p*-Value)
Mesio-Distal	67.96 ± 5.41	65.93 ± 5.71	0.007 *	68.93 ± 6.51	0.727	<0.001 *
Bucco-Lingual	55.48 ± 5.79	49.12 ± 7.47	<0.001 *	52.10 ± 7.79	0.004 *	<0.001 *
*p*-value	<0.001 *	<0.001 *		< 0.001 *		

ISQ: implant stability quotient; T0: implant insertion; T1: 4 months after implant insertion; T2: 6 months after implant insertion; *: statistically significant.

**Table 3:** Marginal bone loss at the different time points.

	Mesial	*p*-Value	Distal	*p*-Value	Mean	*p*-Value
MBL T1-T0	0.38 ± 0.46	<0.001 *	0.40 ± 0.58	<0.001 *	0.38 ± 0.48	<0.001 *
MBL T2-T0	0.43 ± 0.57	<0.001 *	0.60 ± 0.74	<0.001 *	0.60 ± 0.52	<0.001 *
MBL T2-T1	0.09 ± 0.22	<0.001 *	0.20 ± 0.36	<0.001 *	0.20 ± 0.19	<0.001 *

Measurements are expressed in mm as mean ± standard deviation. MBL: marginal bone loss; T0: implant insertion; T1: 6 months after implant insertion; T2: 12 months after functional loading; *: statistically significant.

**Table 4:** Univariate analysis of baseline factors potentially influencing marginal bone loss (MBL).

Variable	MBL (T1−T0) Coeff. [95% CI]	MBL (T2−T0) Coeff. [95% CI]–*p*-Value	MBL (T2−T1) Coeff. [95% CI]–*p*-Value
Gender	0.058 [−0.19/0.31]	−0.065 [−0.34/0.21]	−0.110 [−0.21/−0.11]
Age	−0.006 [−0.02/0.05]	−0.004 [−0.02/0.08]	0.002 [−0.003/0.006]
Smoking	0.018 [−0.19/0.23]	0.032 [−0.18/0.26]	0.017 [−0.07/0.101]
History of Periodontitis	−0.110 [−0.38/0.14]	−0.190 [−0.48/0.09]	−0.042 [−0.15/0.06]
Implant Length	−0.013 [−0.14/0.11]	−0.062 [−0.20/0.08]	−0.047 [−0.09/0.002]
Implant Location	0.283 [−0.02/0.57]	0.322 [−0.009/0.636] *	0.003 [−0.121/0.113]

MBL: marginal bone loss; T0: implant insertion; T1: 6 months after implant insertion; T2: one year of prosthetic loading; Coeff.: coefficient; CI: confidence interval; *: statistically significant.

## References

[1] Cawood J.I., Howell R.A. (1988). A classification of the edentulous jaws. Int. J. Oral Maxillofac. Surg..

[2] Ortega-Oller I., Suárez F., Galindo-Moreno P., Torrecillas-Martínez L., Monje A., Catena A., Wang H.L. (2014). The influence of implant diameter on its survival: A meta-analysis based on prospective clinical trials. J. Periodontol..

[3] Schiegnitz E., Al-Nawas B. (2018). Narrow-diameter implants: A systematic review and meta-analysis. Clin. Oral Implants Res..

[4] Blackburn T.K., Cawood J.I., Stoelinga P.J., Lowe D. (2008). What is the quality of the evidence base for pre-implant surgery of the atrophic jaw?. Int. J. Oral Maxillofac. Surg..

[5] Chappuis V., Rahman L., Buser R., Janner S.F.M., Belser U.C., Buser D. (2018). Effectiveness of contour augmentation with guided bone regeneration: 10-year results. J. Dent. Res..

[6] Orsini G., Stacchi C., Visintini E., Di Iorio D., Putignano A., Breschi L., Di Lenarda R. (2011). Clinical and histologic evaluation of fresh frozen human bone grafts for horizontal reconstruction of maxillary alveolar ridges. Int. J. Periodontics Restor. Dent..

[7] Starch-Jensen T., Becktor J.P. (2019). Maxillary alveolar ridge expansion with split-crest technique compared with lateral ridge augmentation with autogenous bone block graft: A systematic review. J. Oral Maxillofac. Res..

[8] Chappuis V., Cavusoglu Y., Buser D., von Arx T. (2017). Lateral ridge augmentation using autogenous block grafts and guided bone regeneration: A 10-year prospective case series study. Clin. Implant Dent. Relat. Res..

[9] Sanz-Sánchez I., Ortiz-Vigón A., Sanz-Martín I., Figuero E., Sanz M. (2015). Effectiveness of lateral bone augmentation on the alveolar crest dimension: A systematic review and meta-analysis. J. Dent. Res..

[10] Stacchi C., Spinato S., Lombardi T., Bernardello F., Bertoldi C., Zaffe D., Nevins M. (2020). Minimally invasive management of implant-supported rehabilitation in the posterior maxilla, Part II. Surgical techniques and decision tree. Int. J. Periodontics Restor. Dent..

[11] Linkow L.I. (1968). The blade vent—A new dimension in endosseous implantology. Dent. Concepts.

[12] Fritz M.E. (1997). Overview of clinical trials on endosseous implants. Ann. Periodontol..

[13] Noack N., Willer J., Hoffmann J. (1999). Long-term results after placement of dental implants: Longitudinal study of 1,964 implants over 16 years. Int. J. Oral Maxillofac. Implant..

[14] Diotallevi P., Dal Carlo L., Pasqualini M.E., Mazziotti S., Nardone M., Moglioni E. (2014). Radiological evaluation of long term complications of oral rehabilitations of thin ridges with titanium blade implants. J. Osseointegr..

[15] Dal Carlo L., Pasqualini M., Shulman M., Rossi F., Comola G., Manenti P., Candotto V., Lauritano D., Zampetti P. (2019). Endosseous distal extension (EDE) blade implant technique useful to provide stable pillars in the ipotrophic lower posterior sector: 22 years statistical survey. Int. J. Immunopathol. Pharmacol..

[16] Food and Drug Administration H.H.S. (2014). Dental devices; reclassification of blade-form endosseous dental implant. Final order. Fed. Regist..

[17] Koike M., Greer P., Owen K., Lilly G., Murr L.E., Gaytan S.M., Martinez E., Okabe T. (2011). Evaluation of titanium alloys fabricated using rapid prototyping technologies-electron beam melting and laser beam melting. Materials (Basel).

[18] Albrektsson T., Wennerberg A. (2004). Oral implant surfaces: Part 1--review focusing on topographic and chemical properties of different surfaces and in vivo responses to them. Int. J. Prosthodont..

[19] Linkevicius T., Puisys A., Linkevicius R., Alkimavicius J., Gineviciute E., Linkeviciene L. (2020). The influence of submerged healing abutment or subcrestal implant placement on soft tissue thickness and crestal bone stability. A 2-year randomized clinical trial. Clin. Implant Dent. Relat. Res..

[20] Gomez-Roman G., Launer S. (2016). Peri-implant bone changes in immediate and non-immediate root-analog stepped implants-a matched comparative prospective study up to 10 years. Int. J. Implant Dent..

[21] Spray J.R., Black C.G., Morris H.F., Ochi S. (2000). The influence of bone thickness on facial marginal bone response: Stage 1 placement through stage 2 uncovering. Ann. Periodontol..

[22] Chen S.T., Darby I.B., Reynolds E.C. (2007). A prospective clinical study of non-submerged immediate implants: Clinical outcomes and esthetic results. Clin. Oral Implants Res..

[23] Nohra J., Dagher M., Matni G., Mokbel N., Jobaili E., Naaman N. (2018). Effect of primary stability and soft- and hard-tissue thickness on marginal bone loss: A prospective pilot study. Implant Dent..

[24] Pramstraller M., Farina R., Franceschetti G., Pramstraller C., Trombelli L. (2011). Ridge dimensions of the edentulous posterior maxilla: A retrospective analysis of a cohort of 127 patients using computerized tomography data. Clin. Oral Implant. Res..

[25] Katsoulis J., Enkling N., Takeichi T., Urban I.A., Mericske-Stern R., Avrampou M. (2012). Relative bone width of the edentulous maxillary ridge. Clinical implications of digital assessment in presurgical implant planning. Clin. Implant Dent. Relat. Res..

[26] Braut V., Bornstein M.M., Kuchler U., Buser D. (2014). Bone dimensions in the posterior mandible: A retrospective radiographic study using cone beam computed tomography. Part 2—Analysis of edentulous sites. Int. J. Periodontics Restor. Dent..

[27] Donos N., Mardas N., Chadha V. (2008). Clinical outcomes of implants following lateral bone augmentation: Systematic assessment of available options (barrier membranes, bone grafts, split osteotomy). J. Clin. Periodontol..

[28] Kuchler U., von Arx T. (2014). Horizontal ridge augmentation in conjunction with or prior to implant placement in the anterior maxilla: A systematic review. Int. J. Oral Maxillofac. Implant..

[29] Brunski J.B. (1992). Biomechanical factors affecting the bone-dental implant interface. Clin. Mater..

[30] Szmukler-Moncler S., Salama H., Reingewirtz Y., Dubruille J.H. (1998). Timing of loading and effect of micromotion on bone-dental implant interface: Review of experimental literature. J. Biomed. Mater. Res..

[31] Brunski J.B., Moccia A.F., Pollock S.R., Korostoff E., Trachtenberg D.I. (1979). The influence of functional use of endosseous dental implants on the tissue implant interface: I. Histological aspects. J. Dent. Res..

[32] Lum L.B., Beirne O.R., Curtis D.A. (1991). Histological evaluation of HA-coated vs. uncoated titanium blade implants in delayed and immediately loaded applications. Int. J. Oral Maxillofac. Implant..

[33] Rashad A., Sadr-Eshkevari P., Weuster M., Schmitz I., Prochnow N., Maurer P. (2013). Material attrition and bone micromorphology after conventional and ultrasonic implant site preparation. Clin. Oral Implant. Res..

[34] Stacchi C., Berton F., Turco G., Franco M., Navarra C.O., Andolsek F., Maglione M., Di Lenarda R. (2016). Micromorphometric analysis of bone blocks harvested with eight different ultrasonic and sonic devices for osseous surgery. J. Craniomaxillofac. Surg..

[35] Stacchi C., Bassi F., Troiano G., Rapani A., Lombardi T., Jokstad A., Sennerby L., Schierano G. (2020). Piezoelectric bone surgery for implant site preparation compared with conventional drilling techniques: A systematic review, meta-analysis and trial sequential analysis. Int. J. Oral Implantol..

[36] Li J., Yin X., Huang L., Mouraret S., Brunski J.B., Cordova L., Salmon B., Helms J.A. (2017). Relationships among bone quality, implant osseointegration, and Wnt signaling. J. Dent. Res..

[37] Albrektsson T., Chrcanovic B., Östman P.O., Sennerby L. (2017). Initial and long-term crestal bone responses to modern dental implants. Periodontology 2000.

[38] Spinato S., Stacchi C., Lombardi T., Bernardello F., Messina M., Zaffe D. (2019). Biological width establishment around dental implants is influenced by abutment height irrespective of vertical mucosal thickness: A cluster randomized controlled trial. Clin. Oral Implant. Res..

[39] Galindo-Moreno P., León-Cano A., Monje A., Ortega-Oller I., O’Valle F., Catena A. (2016). Abutment height influences the effect of platform switching on peri-implant marginal bone loss. Clin. Oral Implant. Res..

[40] Lombardi T., Berton F., Salgarello S., Barbalonga E., Rapani A., Piovesana F., Gregorio C., Barbati G., Di Lenarda R., Stacchi C. (2019). Factors influencing early marginal bone loss around dental implants positioned subcrestally: A multicenter prospective clinical study. J. Clin. Med..

[41] Spinato S., Stacchi C., Lombardi T., Bernardello F., Messina M., Dovigo S., Zaffe D. (2020). Influence of abutment height and vertical mucosal thickness on early marginal bone loss around implants. A randomized clinical trial with an 18-month post-loading clinical and radiographic evaluation. Int. J. Oral Implantol..

[42] Aloy-Prósper A., Peñarrocha-Oltra D., Peñarrocha-Diago M., Camacho-Alonso F., Peñarrocha-Diago M. (2016). Peri-implant hard and soft tissue stability in implants placed simultaneously versus delayed with intraoral block bone grafts in horizontal defects: A retrospective case series study. Int. J. Oral Maxillofac. Implant..

[43] Meloni S.M., Jovanovic S.A., Urban I., Canullo L., Pisano M., Tallarico M. (2017). Horizontal ridge augmentation using GBR with a native collagen membrane and 1:1 ratio of particulated xenograft and autologous bone: A 1-year prospective clinical study. Clin. Implant Dent. Relat. Res..

[44] Bruschi G.B., Capparé P., Bravi F., Grande N., Gherlone E., Gastaldi G., Crespi R. (2017). Radiographic evaluation of crestal bone level in split-crest and immediate implant placement: Minimum 5-year follow-up. Int. J. Oral Maxillofac. Implant..

[45] Strietzel F.P., Nowak M., Küchler I., Friedmann A. (2002). Peri-implant alveolar bone loss with respect to bone quality after use of the osteotome technique: Results of a retrospective study. Clin. Oral Implant. Res..

[46] Guan H., van Staden R., Loo Y.C., Johnson N., Ivanovski S., Meredith N. (2009). Influence of bone and dental implant parameters on stress distribution in the mandible: A finite element study. Int. J. Oral Maxillofac. Implant..

[47] Kim S., Lee V.J., Lee S., Moon H.S., Chung M.K. (2013). Assessment of pain and anxiety following surgical placement of dental implants. Int. J. Oral Maxillofac. Implant..

[48] Hashem A.A., Claffey N.M., O’Connell B. (2006). Pain and anxiety following the placement of dental implants. Int. J. Oral Maxillofac. Implant..

[49] Pereira G.M., Cota L.O., Lima R.P., Costa F.O. (2020). Effect of preemptive analgesia with ibuprofen in the control of postoperative pain in dental implant surgeries: A randomized, triple-blind controlled clinical trial. J. Clin. Exp. Dent..

[50] Scarano A., Carinci F., Lorusso F., Festa F., Bevilacqua L., Santos de Oliveira P., Maglione M. (2018). Ultrasonic vs drill implant site preparation: Post-operative pain measurement through vas, swelling and crestal bone remodeling: A randomized clinical study. Materials (Basel).

[51] Pistilli R., Felice P., Piattelli M., Nisii A., Barausse C., Esposito M. (2014). Blocks of autogenous bone versus xenografts for the rehabilitation of atrophic jaws with dental implants: Preliminary data from a pilot randomised controlled trial. Eur. J. Oral Implantol..

[52] Payer M., Tan W.C., Han J., Ivanovski S., Mattheos N., Pjetursson B.E., Zhuang L., Fokas G., Wong M.C.M., Acham S. (2020). on behalf of the International Team for Implantology (ITI) Antibiotic Study Group. The effect of systemic antibiotics on clinical and patient-reported outcome measures of oral implant therapy with simultaneous guided bone regeneration. Clin. Oral Implant. Res..

